# Thermoacoustic Curing Mechanism and Process Optimization in Non-Contact Ultrasonic 3D Printing

**DOI:** 10.3390/ma19143019

**Published:** 2026-07-13

**Authors:** Yang Xu, Siqi Yu, Zhiwei Ji, Xiangchen He, Wenlong Xie, Qiangbing Lu, Feng Han, Zheng Li, Guoqing Jin, Minghui Lu

**Affiliations:** 1 School of Mechanical and Electrical Engineering, Soochow University, Suzhou 215100, China; 15255006892@163.com (Y.X.); 20245229076@stu.suda.edu.cn (S.Y.); 20245229049@stu.suda.edu.cn (Z.J.); 20235229042@stu.suda.edu.cn (X.H.); 20235229030@stu.suda.edu.cn (W.X.); 2 Research Institute of Advanced Manufacturing Technology, Soochow University, Suzhou 215100, China; 3 International Institute of Acoustic Technology, Suzhou 215500, China; luqiangbing@iat-center.com (Q.L.); hanfeng@iat-center.com (F.H.); rush666_nju@hotmail.com (Z.L.); 4 College of Engineering and Applied Sciences, Nanjing University, Nanjing 210023, China

**Keywords:** ultrasonic additive manufacturing, thermoacoustic curing, process window, energy localization, Polydimethylsiloxane (PDMS)

## Abstract

Non-contact ultrasonic three-dimensional (3D) printing has emerged as a promising alternative to conventional energy-curing manufacturing techniques. However, an insufficient understanding of its underlying curing mechanisms still limits the precise regulation of solidification behavior and stable structural forming. In this context, this work develops a customized ultrasonic printing platform to investigate the thermoacoustic curing behavior of a modified PDMS-based material system. A multiphysics numerical model is established to characterize the focal acoustic pressure, concentrated energy distribution, and transient temperature evolution during printing. Simulated and experimental results collectively indicate that solidification behavior is governed by the balance between localized acoustic energy confinement and thermal diffusion. Systematic experiments are further conducted to quantify the effects of driving power, scanning speed, and line spacing on forming quality. The optimized parameter combination (17.5 W driving power, 0.4 mm/s scanning speed, and 0.2–0.25 mm line spacing) enables stable single-track morphology and favorable interlayer bonding. Additionally, reciprocating scanning trajectories are found to promote localized thermal accumulation, which may introduce dimensional deviations in printed structures. This study provides insights into ultrasonic thermoacoustic curing behavior and offers feasible process guidance for ultrasonic additive manufacturing under the specific material and experimental configurations adopted in this work.

## 1. Introduction

Conventional layer-by-layer stereolithography and volumetric photopolymerization experience severe light attenuation and scattering in opaque, filler-laden, and multi-component media, which greatly limits the available types of printable inks and restricts the curing penetration depth to the sub-millimeter scale [1]. Two-photon polymerization lithography, despite its capability for nanoscale precision patterning, still induces prominent optical distortion when processing composite materials incorporated with inorganic fillers, further constraining its multi-material manufacturing versatility [2]. As a typical mechanical-forming-based printing technique, fused powder extrusion printing supports diversified material systems but inevitably imposes high shear stress on bioactive components, which precludes in situ fabrication within intact soft biological tissues [3,4]. Such inherent optical and mechanical drawbacks substantially hinder the practical deployment of conventional printing technologies in deep-tissue minimally invasive therapy and opaque composite manufacturing. In contrast, focused ultrasound (FUS)-based ultrasonic 3D printing effectively circumvents the aforementioned technical bottlenecks. Acoustic waves are capable of delivering localized energy at centimeter-scale depths to trigger contact-free crosslinking inside bulk substrates, significantly expanding the portfolio of printable functional polymers for biomedical and advanced structural manufacturing applications [5]. A broad spectrum of 3D-printed acoustic functional devices, ranging from tissue engineering scaffolds to acoustic metamaterials and underwater acoustic lenses, has further verified the huge application potential of acoustic additive manufacturing [6,7,8,9]. Additionally, MRI-derived 3D acoustic-printable models also provide powerful tools for studying coupled acoustic–aerodynamic characteristics of biological structures [10].

A series of landmark studies has validated the feasibility of FUS-driven additive manufacturing. Habibi et al. pioneered direct sound printing (DSP) based on cavitation-initiated PDMS polymerization, whereas the voxel-by-voxel scanning mechanism leads to low manufacturing throughput [11]. To improve fabrication efficiency, Derayatifar et al. proposed holographic acoustic printing that modulates acoustic pressure fields to enable parallel planar curing, achieving remarkable enhancements in forming efficiency and penetration performance [12]. Kuang’s group developed self-enhancing PNIPAm/agar sono-inks for deep-penetration acoustic volumetric printing (DAVP). The thermoresponsive PNIPAm component enhances acoustic absorption while suppressing disruptive acoustic streaming, enabling substrate-free bulk gelation at centimeter-scale depths [13]. Davoodi et al. reported imaging-guided in vivo sound printing (DISP) employing temperature-sensitive liposomes for spatiotemporally controlled crosslinker release, realizing in situ intra-tissue fabrication with a resolution of approximately 150 μm [14]. In addition, continuous sono-thermal curing strategies have been established to enable scalable low-temperature manufacturing of thermoset silicone elastomers [15].

Although remarkable progress has been achieved in hardware development and sono-ink formulation for ultrasonic 3D printing, relevant investigations to date are relatively scattered. Numerous previous works have successfully validated the feasibility of acoustic fabrication and presented prototype printing systems. Nevertheless, few efforts have established systematic quantitative relationships linking core acoustic parameters (frequency, power, scanning speed), rheo-acoustic behaviors of inks, and vital curing performances (penetration depth, dimensional precision, crosslink uniformity). Comprehensive predictive models that facilitate parameter tuning and process window optimization are still scarce, which poses certain challenges to the standardization and industrial deployment of this emerging printing technique.

To address the above research gaps, this study optimizes the fabrication process of non-contact ultrasonic 3D printing through integrated numerical simulation and experimental characterization. A poly(*N*-isopropylacrylamide) (PNIPAm)-modified PDMS–agar composite is selected as the printing material [16]. A custom ultrasonic printing platform is developed, and multiphysics simulations are performed to characterize focal temperature evolution and crosslinking mechanisms. Guided by the thermoacoustic response derived from simulations, systematic experiments quantify the influences of driving power, excitation frequency and scanning velocity on polymerization performance. Combining single-point curing, continuous line fabrication, inter-track spacing screening and full 3D forming trials, this work reveals the underlying thermal and curing behaviors and establishes a robust optimized process window to enable consistent ultrasonic additive manufacturing.

## 2. Construction of the Ultrasonic 3D Printing Platform

To achieve precisely regulated ultrasonic curing, a bespoke printing platform was built by integrating a gantry-type three-axis motion stage and a high-frequency acoustic excitation unit.

**Figure 1:**
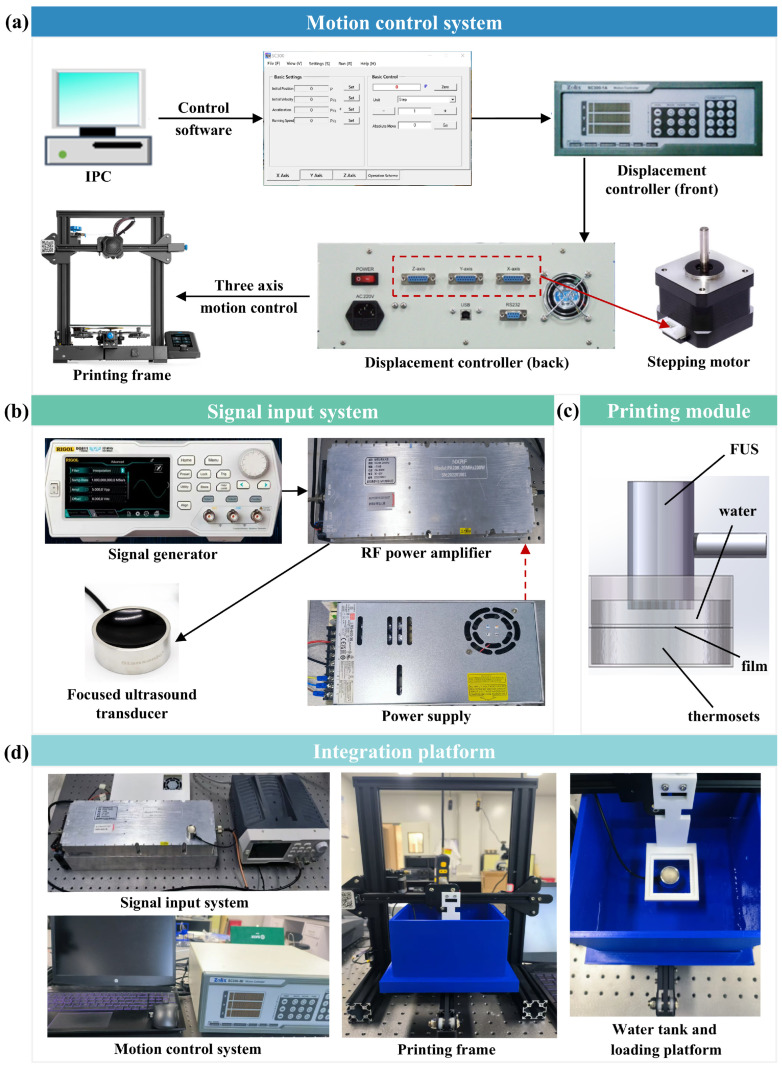
Schematic of the ultrasonic 3D printing system. (**a**) Motion control subsystem that provides programmable three-axis positioning of the transducer assembly; solid red arrows stand for hardware wiring between the displacement controller and stepping motor, and solid black arrows represent digital control signals transmitted to this motion unit. (**b**) Ultrasonic excitation subsystem composed of a signal generator, RF power amplifier, and focused transducer for high-frequency acoustic energy output; solid black arrows denote the signal transmission path among internal excitation devices, while the red dashed arrow indicates the power supply circuit feeding the RF power amplifier. (**c**) Printing unit demonstrating water-coupled FUS-triggered curing and thermosetting resin forming on thin-film substrates. (**d**) Complete integrated printing setup integrating motion control, acoustic excitation unit, printing fixture, water coupling tank, and material carrier platform.

The motion control subsystem (Figure 1a) utilizes a programmable stage controller to deliver micron-scale positioning along three orthogonal directions. Custom software SC300 V1.0 outputs motion instructions that drive synchronized linear actuators, allowing accurate spatial alignment of the ultrasonic focal zone.

The ultrasonic excitation subsystem (Figure 1b) comprises a signal generator, radio-frequency (RF) power amplifier, and focused ultrasound transducer. The generator outputs tunable MHz-frequency waveforms with adjustable frequency and amplitude. Amplified RF signals energize the focused transducer, which transforms electrical input into spatially converged acoustic waves. Acoustic focusing creates a confined high-energy-density zone inside the printing material, triggering localized thermal activation and crosslinking.

The printing assembly (Figure 1c) holds the transducer inside a water-coupled chamber to guarantee efficient acoustic energy transmission. Focused ultrasound travels through the coupling medium and deposits energy inside bulk materials, supporting non-contact curing atop thin-film substrates.

Combining motion control, acoustic excitation and printing assemblies yields the full ultrasonic 3D printing system (Figure 1d). Coordinated spatial positioning alongside tunable acoustic parameters (frequency, output power, sonication duration) enables targeted thermally initiated crosslinking at user-defined coordinates. System calibration and parametric adjustment guarantee reliable performance across diverse feedstocks and processing scenarios.

## 3. Acoustic Energy Deposition and Thermoacoustic Coupling Mechanism

To reveal the energy deposition characteristics of focused ultrasound and its impacts on polymer curing, multiphysics numerical simulations were carried out via COMSOL Multiphysics 5.6. Systematic analyses of the acoustic pressure field, volumetric energy density distribution, and thermoacoustic conversion within the focal zone were performed to elucidate the physical mechanisms governing localized ultrasonic crosslinking.

### 3.1. Numerical Model Configuration

To reveal the energy deposition characteristics of focused ultrasound and its influence on polymer curing, multiphysics numerical simulations were performed using COMSOL Multiphysics. The simulation adopted an axisymmetric computational domain corresponding to the geometry of the concave self-focusing transducer used in the experiments. The computational domain consisted of three regions: the water coupling region, the modified PDMS precursor region, and the focal region around the geometric focus of the transducer. A sinusoidal acoustic pressure boundary condition was applied on the active surface of the transducer to represent ultrasonic excitation, and acoustic continuity conditions were applied at the water–material interface.

The absorbed acoustic energy in the material domain was coupled with transient heat transfer to describe acoustic-to-thermal conversion during localized ultrasonic exposure. The acoustic and thermal parameters assigned to the computational domains are summarized in Table 1. Since the acoustic and thermal properties of the PNIPAm/agar-modified PDMS precursor depend on the additive composition and precursor state, parameter ranges were adopted for the modified PDMS domain. The experimentally applied electrical input power was considered when defining the acoustic excitation level in the simulation to maintain consistency with the ultrasonic printing conditions.

Wavelength-adaptive graded meshes were applied in the simulation, with local refinement in the focal region and near the water–material interface to capture the steep gradients of acoustic pressure and temperature. The numerical results were evaluated at the trend level by comparing the simulated focal energy localization and temperature distribution trends with experimentally observed power-dependent curing behavior, exposure-time-dependent voxel growth, and line-width variation. Therefore, the numerical model was used to support the interpretation of the thermoacoustic curing mechanism and process optimization trends, rather than to provide a direct quantitative prediction of polymer conversion.

### 3.2. Spatial Distribution of Acoustic Pressure and Energy Density

The acoustic pressure field characterizes the amplitude of propagating ultrasonic waves and dictates mechanical stress within materials, while the acoustic intensity field quantifies energy transfer efficiency and directly dominates localized thermal generation. Quantification of the acoustic field’s spatial confinement is achieved by analyzing focal pressure amplitude and intensity distributions across varied excitation frequencies.

**Figure 2:**
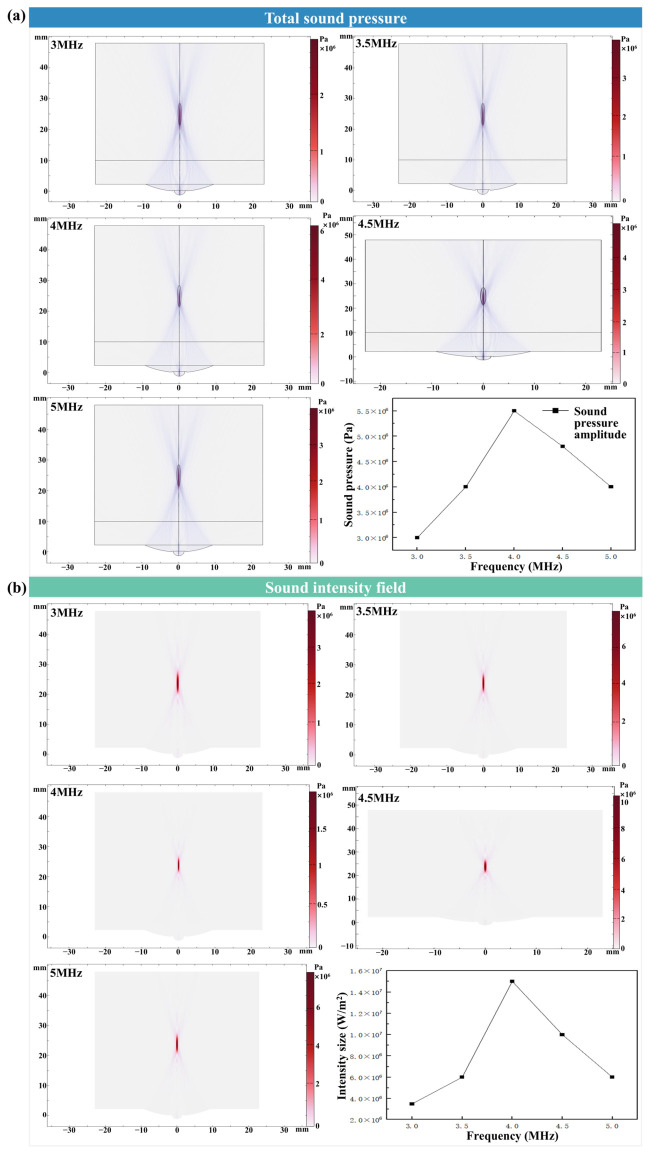
Spatial distributions of acoustic pressure and energy density within the focal zone. (**a**) Simulated full acoustic pressure fields under excitation frequencies ranging from 3 MHz to 5 MHz, along with frequency-dependent peak pressure amplitudes measured at the focal spot. (**b**) Simulated acoustic intensity contours at 3–5 MHz and the corresponding frequency-response curve of focal intensity, revealing the spatial energy localization characteristics.

Raising the excitation frequency from 3 MHz to 5 MHz shortens the acoustic wavelength and strengthens spatial focusing performance. At 3 MHz, focal confinement remains weak, yielding widely dispersed acoustic energy. Elevating frequency sharpens the focus and elevates peak focal pressure and intensity. Nevertheless, excessive frequency intensifies acoustic attenuation and propagation losses, diminishing usable energy delivered to the focal zone (Figure 2a). Acoustic intensity follows a comparable rising-then-falling trend, with more dramatic fluctuations given its quadratic dependence on pressure (Figure 2b). The maximum focal energy output occurs at approximately 4 MHz.

**Figure 3:**
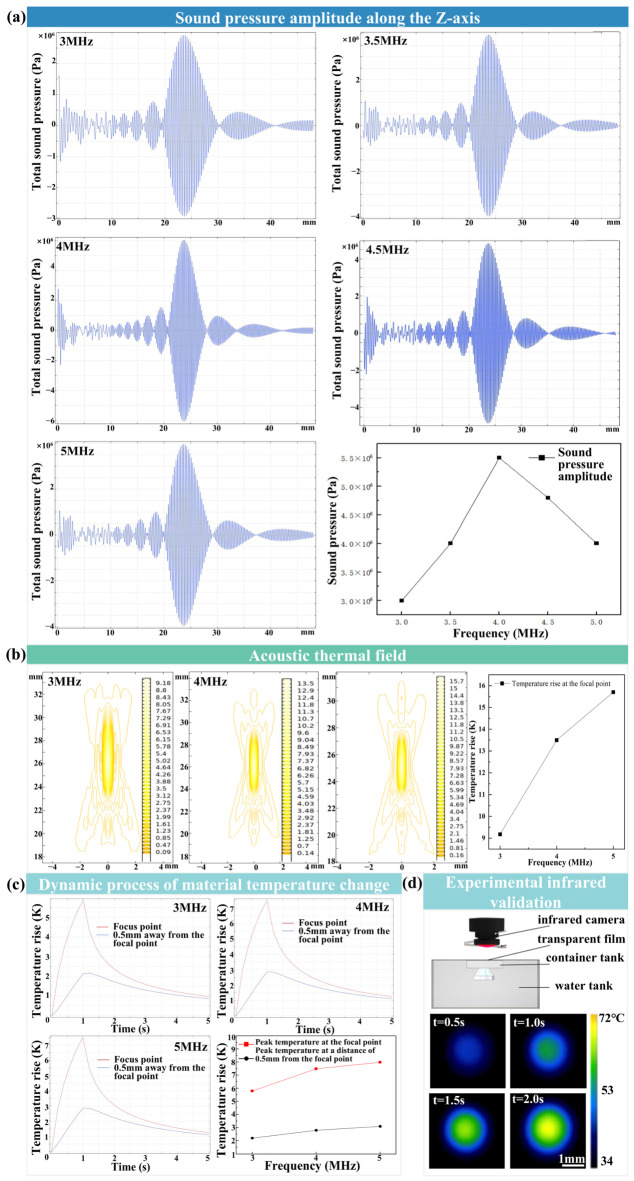
Axial acoustic confinement and experimental validation of thermoacoustic evolution characteristics. (**a**) Axial acoustic pressure amplitude distribution along the *Z*-axis, illustrating focal sharpness and acoustic attenuation performance. (**b**) Simulated temperature rise contours under 1 s ultrasonic irradiation at different excitation frequencies, as well as the transient temperature evolution curves at the focal spot. (**c**) Transient heating and cooling behaviors at the focal plane and the 0.5 mm off-axis position under 1 s ultrasonic exposure across 3–5 MHz frequencies, revealing spatially dependent thermoacoustic dynamic mechanisms. (**d**) Experimental infrared thermal observation of focal heating, including measurement setup and transient temperature evolution images during focused ultrasonic exposure.

Axial pressure amplitude profiles along the symmetry axis (Figure 3a) further quantify focal sharpness and attenuation characteristics. The peak pressure magnitude and energy decay gradient near the focal region jointly describe the spatial localization of acoustic energy, laying a physical foundation for the thermally induced curing reactions discussed below.

### 3.3. Thermoacoustic Coupling and Focal-Region Thermal Evolution

Ultrasonic energy converts to heat via acoustic absorption within the focal zone, inducing localized temperature elevation that triggers material crosslinking once the critical threshold temperature is attained. Transient temperature evolution was numerically solved for three typical excitation frequencies: 3 MHz, 4 MHz and 5 MHz.

Figure 3b displays temperature rise profiles after 1 s of ultrasonic irradiation. Increasing excitation frequency tightens acoustic energy confinement within the focal zone. The 5 MHz case yields the largest focal temperature rise owing to enhanced acoustic absorption, while 3 MHz delivers broadly distributed energy and weaker focal heating. Notably, temperature variations outside the focal area stay marginal, suggesting that ultrasonic 3D printing can achieve spatially selective heating while limiting undesirable thermal diffusion.

At t = 1 s, rapid focal heating drives localized solidification, whereas peripheral regions exhibit milder temperature growth and slower crosslinking kinetics. Extended ultrasonic exposure causes heat buildup and radial thermal spreading, demonstrating the requirement for accurate temporal regulation to constrain the curing area.

Thermoacoustic dynamic behaviors were further analyzed by comparing temperature evolutions at the focal plane and an off-axis point 0.5 mm away (Figure 3c). The focal zone undergoes fast temperature ramping with a higher peak value and rapid cooling once excitation terminates. In comparison, the off-axis position features slow, moderate heating, a lower temperature peak and gentler cooling responses. Such discrepancies stem from the spatial gradient of acoustic energy density.

Systematic comparative analysis identifies 4 MHz as the optimal operating frequency. The 3 MHz configuration has inadequate focusing and poor energy confinement; conversely, 5 MHz exhibits superior focusing yet severe acoustic attenuation that undermines effective energy delivery. The 4 MHz condition strikes a favorable balance between focal sharpness and energy transfer efficiency, generating sufficient acoustic pressure and thermally activated crosslinking to enable rapid material curing. This optimal frequency provides a reasonable guideline for parameter configuration in ultrasonic additive manufacturing.

To further support the simulated thermoacoustic response, infrared thermal imaging experiments were conducted under focused ultrasonic excitation. As shown in Figure 3d, a localized temperature-rise region was experimentally observed around the acoustic focus during ultrasonic exposure. The experimentally observed focal heating behavior agrees with the simulated temperature localization characteristics, providing additional evidence for the thermoacoustic conversion process involved in ultrasonic curing.

### 3.4. Energy Localization and Curing Threshold Mechanism

Curing behavior during ultrasonic 3D printing is dominated by localized acoustic energy deposition and subsequent thermal conversion within the focal volume. Simulation results demonstrate that acoustic intensity governs the rate of temperature rise via acoustic absorption. Polymerization is initiated once the transient temperature surpasses the curing threshold of the polymer material, while inadequate energy input results in incomplete or unstable voxel formation.

The spatial confinement of ultrasonic curing is governed by the competitive interplay between acoustic focusing and thermal diffusion. Acoustic focusing concentrates acoustic energy within a confined zone, whereas thermal diffusion gradually dissipates heat to the surrounding regions. At a low frequency of 3 MHz, insufficient acoustic focusing leads to dispersed energy deposition and poor curing localization. By contrast, the 5 MHz condition delivers tighter focusing but experiences severe acoustic attenuation, which compromises effective energy transmission. The intermediate frequency of 4 MHz achieves an optimal trade-off between focal intensity and energy transfer efficiency, enabling rapid localized heating while suppressing off-axis thermal diffusion.

This threshold-dependent energy effect elucidates the parametric dependence of printed voxel dimensions and line widths on ultrasonic excitation conditions. Stable and high-precision printing requires the input of acoustic energy to adequately exceed the polymerization threshold, while restricting excessive thermal diffusion outside the predefined curing region.

## 4. Parameter-Dependent Curing Dynamics and Structural Formation

Guided by the energy-threshold model, systematic experiments were performed to investigate the effects of excitation parameters on curing morphology and dimensional accuracy at the single-voxel, linear-track, and macroscopic structural scales.

### 4.1. Localized Voxel Formation Under Focused Ultrasound

Single-point curing experiments were performed to investigate voxel formation behavior under focused ultrasound excitation [17,18]. A TQ20-4020 focused transducer (Beijing Orient Jinrong Ultrasonic Circuit Co., Ltd., Beijing, China) with a resonant frequency of 4.158 MHz was used in a thermostatted water bath at 25 °C. The input power varied from 10 W to 25 W, and the exposure duration was adjusted to evaluate the effects of acoustic energy deposition and curing time on voxel formation. Each condition was repeated four times, and the average dimensional measurements were used for analysis.

A PNIPAm/agar-modified PDMS composite was adopted as the printing material. The composite was fabricated based on commercial Sylgard 184 PDMS (Dow Corning Corporation, Midland, MI, USA) with a base-to-curing-agent mass ratio of 10:1. The modified precursor consisted of an 85 wt% Sylgard 184 base component, 8.5 wt% curing agent, 3.5 wt% PNIPAm, and 3 wt% agar, relative to the total precursor mass. PNIPAm and agar solutions were gradually introduced into the PDMS precursor, followed by ultrasonic dispersion and vacuum degassing before printing. This preparation process reduced trapped bubbles and minimized potential disturbances to acoustic propagation during focused ultrasonic exposure. The incorporation of PNIPAm and agar was considered to contribute to acoustic energy absorption and thermoacoustic conversion during focused ultrasonic excitation.

#### 4.1.1. Power-Dependent Voxel Growth

**Figure 4:**
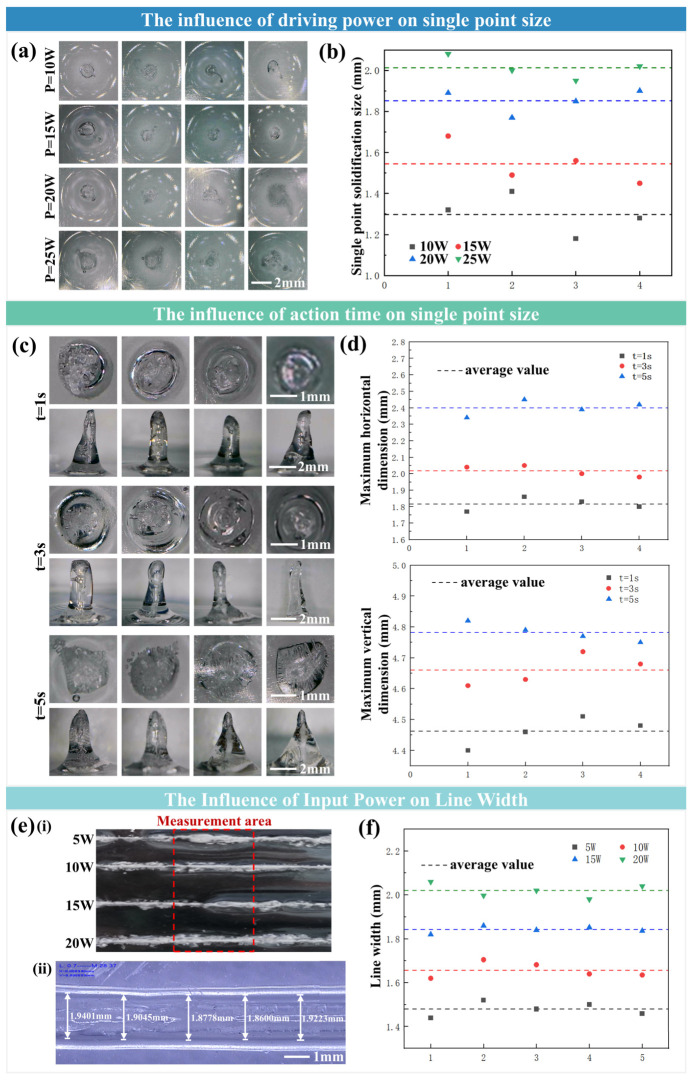
Parameter-dependent voxel and line formation behaviors. (**a**) Representative voxel morphologies fabricated under different driving powers. (**b**) Quantitative variations in voxel dimensions as a function of input power. (**c**) Temporal voxel formation characteristics under varied ultrasonic exposure durations. (**d**) Evolution of lateral width and vertical height with increasing exposure time. (**e**) Power-dependent single-line printing morphologies and the corresponding width measurement strategy: (**i**) Printed lines fabricated at different input powers, where the stable central solidified region was selected as the measurement zone; (**ii**) Five-point sampling method adopted to quantify the line width within the designated measurement region of a single solidified line. (**f**) Statistical distribution of printed line widths obtained from five replicate tests.

The experimental results (Figure 4a,b) verify a minimum power threshold of approximately 10 W, below which effective curing cannot be initiated. A stable and uniform voxel morphology is obtained at an input power of 15 W. When the power exceeds 20 W, the voxel size continuously increases, accompanied by over-curing behavior and morphological defects. This phenomenon indicates that thermal diffusion dominates the curing process and breaks the optimal spatial confinement. Once the curing threshold is satisfied, voxel dimensions scale positively with acoustic intensity until the energy transfer limit of the printing system is reached.

#### 4.1.2. Exposure-Time-Controlled Curing Expansion

At a fixed power of 15 W, prolonged ultrasonic exposure induces continuous growth in both lateral and vertical voxel dimensions (Figure 4c,d) owing to cumulative thermal energy deposition. When the exposure duration exceeds 3 s, pronounced lateral expansion emerges near the substrate, generating uneven curing morphology driven by heat accumulation and induced temperature gradients. A processing window with 15–20 W input power and 3 s ultrasonic exposure enables stable, spatially confined voxel formation.

The observed voxel evolution is consistent with the thermoacoustic heating behavior indicated by the infrared thermal observation in Figure 3d. Localized acoustic energy absorption generates a confined thermal field within the modified PDMS precursor, enabling curing once the energy threshold is reached. Insufficient input power leads to incomplete curing due to inadequate energy deposition, whereas prolonged exposure induces cumulative thermal accumulation and diffusion, resulting in voxel enlargement and lateral spreading. Therefore, stable voxel formation depends on maintaining an appropriate balance between localized energy deposition and suppression of diffusion-driven over-curing.

### 4.2. Continuous Line Formation and Resolution Control

Single-line printing experiments were carried out to characterize the dynamic energy confinement behavior during transducer scanning. Linear printing tests with varying input power and scanning speed were performed in five parallel replicates. For each stable printed track, five equidistant positions were measured, and the average line width was calculated to ensure experimental data reliability.

#### 4.2.1. Power-Modulated Line Width

Under a fixed excitation frequency of 4.158 MHz, the input power was tuned from 5 W to 20 W to investigate line-printing performance (Figure 4e,f). Discontinuous printed lines were obtained at power levels ≤15 W, which stemmed from inadequate acoustic energy input. In contrast, power inputs ≥20 W induced intense acoustic streaming and aggravated thermal diffusion, leading to widened line features and degraded dimensional stability. A stable and uniform linear morphology was achieved within the power range of 15–20 W, which well matches the optimal energy localization process window determined in voxel-scale experiments.

#### 4.2.2. Scanning-Speed-Dependent Stability

**Figure 5:**
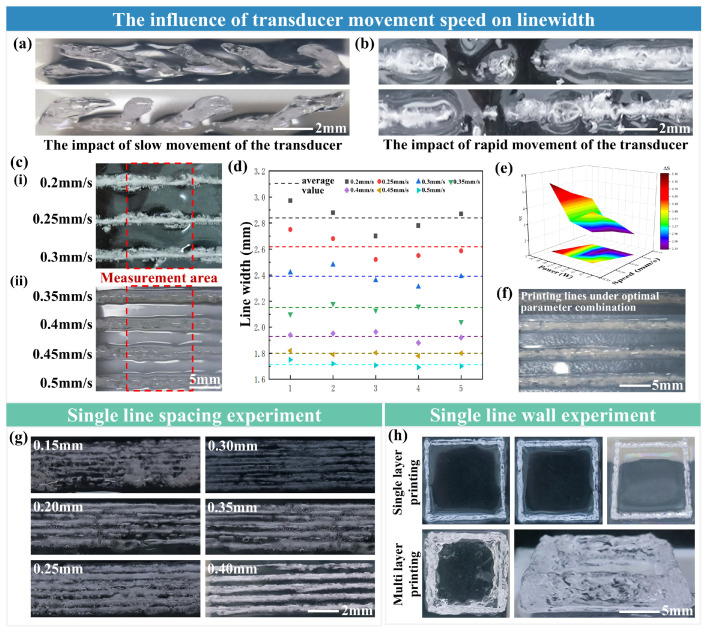
Scanning-speed-dependent line formation and structural stability. (**a**) Line morphology obtained at low scanning speed, exhibiting excessive vertical material accumulation. (**b**) Line morphology obtained at high scanning speed, showing incomplete and non-uniform curing features. (**c**) Speed-induced evolution of line formation: (**i**) irregular and discontinuous curing induced by insufficient energy deposition; (**ii**) uniform line width achieved within the stable processing window. (**d**) Schematic diagram of line width evaluation within the designated measurement area. (**e**) 3D response surface illustrating the combined effects of driving power and scanning speed on dimensional error ΔS. (**f**) Representative line morphology printed under the optimal processing parameter combination. (**g**) Line deposition characteristics under varied inter-line spacing conditions. (**h**) Structural morphologies of single-layer and multilayer vertical printing.

At a fixed excitation frequency of 4.158 MHz, scanning speeds, ranging from 0.2 mm/s to 0.5 mm/s, were systematically investigated (Figure 5a–d). Scanning speeds lower than 0.3 mm/s induce over-curing defects owing to a prolonged energy residence time. In contrast, a moderate speed range of 0.4–0.5 mm/s produces well-defined, uniform line morphologies. Further increasing the scanning speed shortens the ultrasonic interaction time, thereby causing insufficient energy deposition and incomplete curing.

To further optimize the processing window, factorial experimental optimization was conducted by tuning the input power from 15 W to 20 W at 2.5 W intervals and the scanning speed from 0.4 mm/s to 0.5 mm/s at 0.05 mm/s intervals. To quantitatively assess the dimensional accuracy of printed structures, ΔS, defined as the average dimensional error percentage of printed samples, was adopted as the evaluation indicator based on repeated multi-dimensional measurements. A total of three power levels (15 W, 17.5 W, and 20 W) and three scanning speeds (0.4 mm/s, 0.45 mm/s, and 0.5 mm/s) were investigated systematically (Table 2, Table 3 and Table 4). Range analysis results reveal that input power (R = 2.11) imposes a more significant effect on dimensional accuracy compared with scanning speed (R = 1.37). The minimum ΔS value was achieved at the optimal combination of 17.5 W input power and 0.4 mm/s scanning speed (Figure 5e,f).

### 4.3. Inter-Line Thermal Interaction

Line spacing is a critical parameter that directly governs the structural continuity of printed patterns. A line spacing smaller than 0.2 mm causes excessive material overlap between adjacent tracks, while spacings exceeding 0.3 mm induce inter-line gaps and structural discontinuities. The optimized line spacing, ranging from 0.2 mm to 0.25 mm, effectively eliminates severe overlap and achieves uniform material deposition (Figure 5g), which is consistent with the spatial dimension of the curing zone predicted by numerical simulations.

### 4.4. Multi-Layer Accumulation and Structural Integrity

A vertically stacked single-line wall with a side length of 20 mm was fabricated to evaluate the interlayer structural stability of ultrasonic printing (Figure 5h). At rectangular corners, trajectory deceleration prolongs ultrasonic exposure time, causing localized heat accumulation and intensified curing that result in undesirable regional thickening. Such cumulative thermal effects demonstrate that the structural stability of multilayer printing relies on precise maintenance of localized acoustic energy confinement and effective suppression of diffusion-dominated over-curing behavior.

## 5. Structural-Scale Validation of the Energy-Threshold Framework

Following systematic validations at the voxel and line scales, full 3D fabrication was conducted to verify the feasibility of the energy-threshold coupling mechanism under practical path-planned solid printing scenarios.

**Figure 6:**
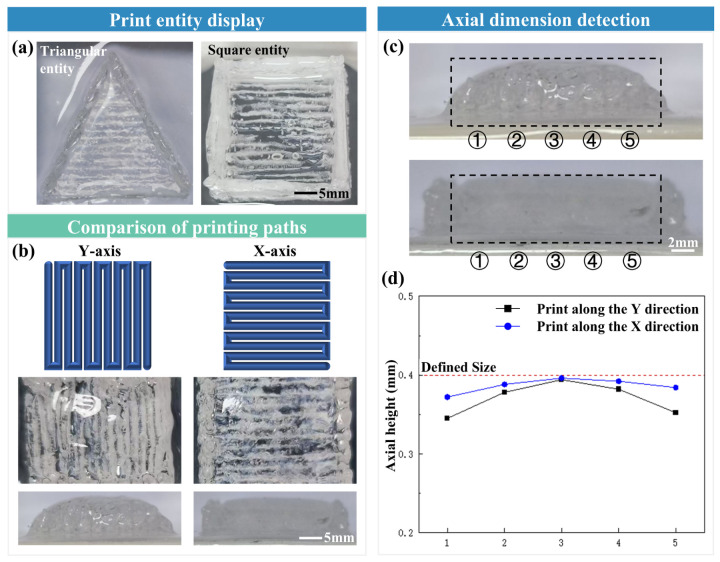
Structural-scale fabrication and path-dependent dimensional variation. (**a**) Representative triangular and square solid structures printed with optimized processing parameters. (**b**) Morphological comparison of samples fabricated via *X*-axis and *Y*-axis unidirectional scanning. (**c**) Schematic of the axial dimensional evaluation strategy for structures printed with different scanning paths; the dashed box denotes the measurement range, and the numbered marks ①–⑤ correspond to the five sampling points of the five-point measurement method. (**d**) Quantitative comparison of axial height variations between X-direction and Y-direction printed samples.

Representative solid structural models were fabricated using the optimal process parameters determined in Section 4 (Figure 6a). Beyond basic printing feasibility verification, this experiment further explores the dimensional stability and energy confinement behavior occurring during volumetric deposition.

To explore the effects of printing path on energy distribution, samples were fabricated using X-direction and Y-direction scanning strategies (Figure 6b). Both scanning modes produced structures with well-matched in-plane dimensions relative to the design model, while slight morphological differences were observed along the *Z*-axis. Y-scanned samples tended to form a slightly convex profile with higher centers and lower edges, whereas X-direction scanning contributed to a more uniform vertical height distribution. This observation suggests that scanning direction may affect axial energy accumulation and interlayer thermal uniformity during multilayer ultrasonic 3D printing.

To quantitatively characterize such directional differences in vertical morphology, a five-point sampling strategy was adopted for axial dimension measurement, with five independent printing replicates for each group (*n* = 5), as depicted in Figure 6c. The statistical results in Figure 6d reveal that the Y-scanned samples exhibit a lower average vertical height (0.37 mm) compared with their X-scanned counterparts (0.39 mm). Standard deviations derived from the five parallel tests were calculated to quantify the experimental measurement uncertainty.

Mechanistically, Y-direction scanning involves frequent reciprocating passes across the structural center, leading to iterative acoustic energy deposition and progressive thermal accumulation. This localized heat buildup produces a central axial height deviation of approximately 10%, resulting in non-uniform vertical profiles. In contrast, X-direction scanning enables a more homogeneous distribution of acoustic energy along the printing path, mitigating excessive thermal accumulation and thereby maintaining stable and consistent axial dimensional uniformity.

These findings suggest that, at the structural scale and for the simple square and triangular geometries investigated herein, printing path planning influences spatial energy distribution and cumulative thermal effects under the present process conditions. The results further indicate that stable solid fabrication depends not only on optimized power and scanning speed parameters, but also on appropriate trajectory strategies that maintain localized energy deposition within the threshold-controlled curing regime.

## 6. Conclusions

This study presents a physics-driven framework for non-contact ultrasonic 3D printing based on localized acoustic energy deposition and threshold-modulated thermoacoustic curing. Combined multiphysics simulations and systematic experimental characterization indicate that curing behavior is mainly associated with the competitive balance between acoustic energy confinement and thermal diffusion. Specifically, the spatial distribution of acoustic pressure and intensity determines the focal energy density, while transient thermal evolution affects whether the localized material reaches the critical curing threshold within a confined volumetric region. This energy-threshold relationship helps explain the observed printing behaviors, including voxel formation characteristics, line-width variation, inter-line overlap defects, and multilayer structural stability under the investigated excitation conditions.

An optimized processing window was determined under the fixed conditions of printing material, ultrasonic transducer, water bath environment, and substrate. The optimal parameter combination, including a driving power of 17.5 W, a scanning speed of 0.4 mm/s, and a line spacing of 0.2–0.25 mm, enabled stable energy localization and reduced diffusion-dominated over-curing. Further structural-scale experiments suggest that scanning trajectory planning can influence cumulative energy redistribution. Path-dependent thermal accumulation, particularly during reciprocating scanning motions, may affect axial dimensional uniformity. These findings indicate that the printing resolution and structural integrity of ultrasonic 3D printing are influenced not only by power and scanning speed, but also by scanning motion strategies.

Despite the above progress, several inherent physical limitations still restrict printing performance. The achievable spatial resolution is constrained by the acoustic wavelength and focusing properties of the transducer. Long-duration energy input can induce cumulative thermal effects, posing challenges to precise dimensional control. In addition, the library of printable materials compatible with thermoacoustic curing requires further expansion. Future research can focus on adopting high-frequency micro-transducer arrays to enhance spatial energy confinement, developing integrated thermal regulation strategies to mitigate heat accumulation, and exploring functional polymer composites and biocompatible material systems to broaden the application scope of ultrasonic 3D printing.

In summary, ultrasonic 3D printing can be regarded as a threshold-regulated energy localization manufacturing process. By controlling the interplay between acoustic energy confinement and thermal diffusion, this technique enables selective volumetric curing in a non-contact manner. The thermoacoustic characteristics identified in this work may provide theoretical insights and technical references for advanced manufacturing applications that require internal structural regulation, including tissue engineering scaffolds, acoustic metamaterials, and flexible functional devices. Further optimization of energy field engineering and scanning trajectory programming may improve the fabrication precision and processing capability of ultrasonic additive manufacturing.

## Figures and Tables

**Table 1:** Acoustic and thermal parameters assigned to the computational domains.

Material/ Domain	Density (kg m^−3^)	Sound Speed (m s^−1^)	Acoustic Attenuation Coefficient (Np m^−1^ MHz^−1^)	Specific Heat Capacity (J kg^−1^ K^−1^)	Thermal Conductivity (W m^−1^ K^−1^)
Water coupling region	1000	1480–1520	0.025	4180–4200	0.60
Modified PDMS precursor region	970–1000	1000–1300	0.10–0.15	1300–1500	0.15–0.22

**Table 2:** Table of influencing factors.

Influence Factor	Level		
	1	2	3
Power (W)	15	17.5	20
Speed (mm/s)	0.4	0.45	0.5

**Table 3:** Two-factor three-level complete experiment.

Number	Power	Speed	Dimensional Error			
	(W)	(mm/s)	ΔX	ΔY	ΔZ	ΔS
1	15	0.4	3.77	1.05	2.23	2.35
2	15	0.45	3.62	2.38	4.2	3.4
3	15	0.5	4.52	3.67	4.23	4.14
4	17.5	0.4	2.52	1.23	2.89	2.21
5	17.5	0.45	2.24	3.21	1.69	2.38
6	17.5	0.5	3.04	2.82	3.92	3.26
7	20	0.4	3.98	4.42	3.96	4.12
8	20	0.45	4.72	5.13	4.1	4.65
9	20	0.5	5.82	6.84	3.54	5.4

**Table 4:** Range analysis table.

Influence Factor	ΔS (%)	
	Power	Printing Speed
K1	9.89	8.68
K2	7.85	10.43
K3	14.17	12.8
k1	3.29	2.89
k2	2.62	3.48
k3	4.72	4.27
R	2.11	1.37

## Data Availability

The original contributions presented in this study are included in the article. Further inquiries can be directed to the corresponding authors.
